# Translating predictive models into clinical practice: fast-and-frugal trees for postoperative delirium using routine data

**DOI:** 10.1038/s41598-026-47452-3

**Published:** 2026-04-18

**Authors:** Odette Wegwarth, Felix Balzer, Sebastian D. Boie, Niklas Giesa, Anika Müller, Jan K. Woike, Claudia Spies, Helge Giese

**Affiliations:** 1 Department of Anesthesiology and Intensive Care Medicine | CCM | CVK, Heisenberg Chair for Medical Risk Literacy & Evidence-Based Decisions, Charité–Universitätsmedizin Berlin, 10117 Berlin, Germany; 2 Center for Adaptive Rationality, Max Planck Institute for Human Development, Berlin, Germany; 3 Institute of Medical Informatics, Charité – Universitätsmedizin Berlin, Berlin, Germany; 4 Department of Anesthesiology and Intensive Care Medicine | CCM | CVK, Charité–Universitätsmedizin Berlin, Berlin, Germany; 5 School of Psychology, University of Plymouth, Plymouth, UK

**Keywords:** Health care, Medical research, Risk factors

## Abstract

Postoperative delirium (POD) is a frequent and serious complication associated with increased morbidity and mortality. Although predictive models for POD exist, they are rarely implemented in routine clinical practice, partly due to their complexity and data requirements. Simple and interpretable tools based on routinely available data may improve clinical applicability. In this retrospective cohort study, we evaluated Fast-and-Frugal Trees (FFTrees) for predicting POD using routine electronic health record data from 61,150 surgical patients treated between 2017 and 2020 at a large German university hospital. We assessed the transferability of previously developed FFTrees and compared their performance with logistic regression, classification and regression trees, random forests, and support vector machines. Updated FFTrees were derived for pre- and peri-operative prediction. Previously published FFTrees showed reduced performance in routine data but improved after recalibration. Updated FFTrees achieved balanced accuracies of 58% (pre-operative) and 60% (peri-operative), with performance comparable to more complex models. Although predictive accuracy was moderate across all models, FFTrees offer advantages in simplicity, transparency, and ease of implementation. As easily memorized decision rules, they can help clinicians organize familiar cues in an optimal order, supporting delirium risk assessment in routine practice where more complex tools are rarely used.

**Supplementary Information:**

The online version contains supplementary material available at 10.1038/s41598-026-47452-3.

## Introduction

Postoperative delirium (POD)—an acute disturbance of attention, awareness, and cognition following surgery—is a common and serious complication, particularly in older and medically complex patients. POD is associated with prolonged hospital stays, increased risk of institutionalization, higher readmission rates, and increased mortality^1,2^. Early identification of patients at elevated risk is therefore essential, as preventive strategies implemented before or during surgery can reduce delirium incidence and improve patient outcomes^3^.

Numerous predictive models have been developed to identify patients at risk for POD^4–9^, consistently highlighting factors such as advanced age, higher American Society of Anesthesiologists (ASA) physical status, comorbidity burden, and impaired cognitive or functional status^10^. However, despite these advances, routine implementation of POD risk prediction remains limited. Existing tools are often underused, even when guidelines recommend systematic screening and prevention strategies^3,11,12^.

A key reason for this implementation gap is the complexity of many prediction tools. Many models require extensive data collection, composite scoring, or computational support, limiting their use in time-constrained clinical workflows. Moreover, guidelines often identify relevant risk factors but provide limited guidance on how to combine them into a structured assessment in routine practice^10^.

Fast-and-frugal decision trees (FFTrees) offer a potential alternative. These simple heuristic models rely on a small number of sequential cues and allow decisions to be made after evaluating only limited information^13,14^. Their transparent, rule-based structure makes them easy to interpret, memorize, and apply without computational tools, which may facilitate implementation in routine clinical settings.

Previous work by Heinrich et al.^6^ demonstrated that FFTrees can predict POD risk using structured research data. However, models developed in controlled datasets often do not translate well to routine clinical environments, where predictors may be incompletely documented or unavailable. As a result, performance may decline when applied to real-world electronic health record (EHR) data.

The present study therefore evaluates whether FFTrees remain applicable under these conditions. Using routine clinical data from more than 61,000 surgical patients, we (1) assess the transferability of previously developed FFTrees, (2) compare their performance with commonly used machine-learning approaches, and (3) develop updated FFTrees optimized for routine data. By focusing on both performance and usability, this study examines whether simple, transparent models can support the practical implementation of POD risk assessment in everyday clinical practice.

## Methods

The previous version of the manuscript has been posted on the Open Science Framework preprint server^4^.

### Ethics

Ethical approval for this study (Ethical Committee Nr. EA4/254/21) was provided by the Ethical Committee of Charité –Universitätsmedizin Berlin, Germany. All methods were performed in accordance with the relevant guidelines and regulations. All data used in this retrospective analysis were originally collected during routine clinical care and not specifically for research purposes. Patients provided general informed consent for the anonymized use of their data for research purposes, as part of the formal in-hospital treatment contract and in accordance with institutional policies.

### Inclusion of participants

To cross-validate existing FFTrees, compare algorithmic performance, and update FFTree models for practical clinical use, we extracted routine clinical data from electronic health records for patient admissions between January 1, 2017, and December 31, 2020, across three campuses of Charité – Universitätsmedizin Berlin (Germany) [more details, see 5]. Adult patients who were admitted to one of the three campuses at Charité to receive surgery and had received at least one Nursing Delirium Screening Scale (Nu-DESC) score in the recovery room were included in the dataset (see 5]). Patients with cardiovascular or craniotomy surgeries were deliberately excluded from analysis as they do not generalize to the broader surgical patient population due to differences in delirium pathophysiology^1^. These patients are consistently considered to be at uniformly high risk for postoperative delirium and are therefore typically managed with standardized, resource-intensive monitoring and prevention protocols, including routine delirium screening and intensive care surveillance. As a result, risk stratification tools are less likely to influence clinical decision-making in these settings. The present study instead focuses on patient populations in which delirium risk is less clearly defined and where practically applicable risk stratification is required to guide the allocation of limited monitoring resources, as not all patients can undergo intensive postoperative surveillance. In such contexts, simple predictive models may be particularly valuable for identifying intermediate-risk patients in whom targeted monitoring and preventive interventions can be prioritized.

### Assessment of POD and measures for the models

Patients were considered POD negative if their Nu-DESC score in the recovery room was equal to 0. If a patient’s Nu-DESC score was 1 or higher, they were considered at least subsyndromal delirious.

The aim was to test the performance of FFTrees for the pre-operative setting based on existing pre-operative cues (e.g., age, body mass index, certain health deficits) and for the peri-operative setting with data that also considered peri-operative cues (duration of anesthesia and duration of surgery). Five types of information were available in the routine clinical dataset: demographics, ICD-10 coded pre-existing medical conditions, surgery information, rated scores, and recoded scores.

*Demographics*: Age, body mass index, weight, height, sex, drinking behavior, history of smoking behavior, and history of Nu-DESC diagnosed POD were included in the routine clinical dataset.

*ICD-10 coded pre-existing medical conditions*: Treating personnel coded the following ICD diagnoses: alcohol related disorders, amputation, anemia, anxiety disorder, acute respiratory distress syndrome (ARDS), carcinoma, cerebral infarction, chronical kidney disease, chronical pain, cognitive impairment, chronic obstructive pulmonary disease (COPD), coronary heart disease, dehydration, dementia, depression, diabetes mellitus, dissociative disorder, drug related disorder, heart failure, HIV, hypercholesterolemia, hyperlipidemia, hypertension, infection diagnosis, liver cirrhosis, metabolic acidosis, nicotine related disorders, nutritional deficiency, obesity, Parkinson’s disease, peripheral arterial disease, peripheral vascular disease, psychiatric disorder, respiratory failure, sleep apnea, and transient ischemic attack (TIA). The known history of the same diagnoses was also included, as was the number of all pre-existing conditions.

*Surgery information*: Surgery billing codes were extracted, including information on the type and location of surgery (blood vessels, digestive tract, facial organs, hearing organs, hormone system, lymphatic system, locomotor organs, nervous system, olfactory organs, parturition, reproductive organs, respiratory tract, skin, urinary system, and visual organs). Peri-operative information—duration of anesthesia and surgery—was also included.

*Rated scores*: The routine clinical dataset al.so contained the following scores supplied by the treating personnel: American Society of Anesthesiologists Physical Status Classification System (ASA status), Barthel index, frailty index, Glasgow Coma Scale (GCS), Mini-Cog^15^, nutritional risk screening (NRS), operative national classifications, pain numeric rating scale, Richmond Agitation–Sedation Scale (RASS), and Sequential Organ Failure Assessment (SOFA).

*Recoded Scores*: CCI^16^ and surgery site—both meaningful cues in the original FFTrees built by Heinrich et al.^6^—were not available in the routine clinical dataset. To create surrogates, we used information on ICD-10 coded pre-existing medical conditions and their history to code CCI. In accordance with the approach of Heinrich et al.^6^, age information—which is part of the CCI—was not included; age was already considered in a separate cue. Surgery codes were used to determine whether a surgery was performed peripherally or not.

### Analyses

#### Transferability of existing FFTrees

In order to investigate the transferability of the pre-operative and peri-operative FFTree models established by Heinrich et al.^6^ to the new routine clinical dataset, the balanced accuracy (i.e., the mean of sensitivity and specificity) of both models were assessed using two distinct approaches: first, by applying the original tree structure and cues with the exact same thresholds proposed by Heinrich et al.^6^ to the new routine clinical dataset without any modification; and second, by using the originally proposed structure and cues but updating the thresholds of the cues to optimize balanced accuracy in the routine clinical dataset^14^.

#### Comparison of algorithm performances

We investigated whether the dfan and ifan algorithms for FFTree construction would perform as well in the routine clinical dataset as they did in the research dataset^6^. To this end, we newly trained and tested both algorithms with the routine clinical dataset and compared them to the balanced accuracy performance of logistic regression, classification and regression tree (CART), random forest, and support vector machine (SVM) algorithms as implemented in the FFTree package^14^ for both the pre-operative and the peri-operative contexts. FFTree algorithms were allowed to use a maximum of five cues and were set to maximize balanced accuracy. In order to estimate the predictive performance, the dataset was randomly split into training and test sets 100 times, each time with the proportions of 80% training data and 20% test (prediction) data. Both dfan and ifan FFTree and the comparator algorithms estimated models based on the training set, and their performance was assessed based solely on the test (prediction) set.

#### Updating FFTrees for use in routine clinical data

Based on the performance in the algorithm comparison and because the dfan algorithm adjusts for dependencies in the predicting cues, we eventually choose the dfan algorithm with balanced accuracy optimization to update the pre-operative and perioperative FFTree models using the full routine clinical dataset (*n* = 61,150).

#### Data handling

We used mean replacement for continuous missing information and the mode for all categorical missing data. Information on the degree of missingness can be obtained from the original publication of this dataset^5^. Generally, only existent surgery codes and medical conditions were coded; they were considered non-applicable (0) unless existent. The models and algorithms for the pre-operative setting used only variables that could be known prior to surgery. The models and algorithms for the peri-operative setting also used all available cues apparent during but not cues that could have been only available after surgery. In order to make it easier to implement the FFTree models in clinical routine, medical conditions and surgery codes were entered as binary factors.

#### Evaluation of model performance

To assess model performance, we report sensitivity, specificity, positive predictive value (PPV), negative predictive value (NPV), accuracy, and balanced accuracy, each with bootstrapped 95% confidence intervals. Differences between models were primarily interpreted based on these confidence intervals. While formal pairwise statistical tests (e.g., DeLong test) could be applied, the focus of this study was on clinically interpretable performance metrics at fixed decision thresholds rather than hypothesis testing of model differences. All analyses were run in R (version 4.2.2).

## Results

### Participant description

Overall, data from 61,150 patients with their last recorded surgery (54% female; Mean[M]_age_ = 52.9 years, Standard Deviation [SD]_age_ = 18.6 years; 9% POD positive) were considered for analyses (more details, see^5^. Unlike the previously published information by Giesa et al., which reported a sample of *N* = 61,187, we excluded 37 participants due to negatively recorded anesthesia durations, leaving a total of 61,150.

### Transferability of existing FFTrees

The pre-operative FFTree established by Heinrich et al.^6^ achieved a balanced accuracy of 49% in the routine clinical dataset, increasing to 54% after recalibration of cue thresholds (CCI without age > 0, frailty > 1; Table 1). The peri-operative FFTree achieved a balanced accuracy of 53%, which improved to 59% after threshold adjustment (anesthesia duration > 112 min).

**Table 1:** Performance of FFTrees originally developed using research data when applied to the routine clinical dataset.

Model	Sensitivity	Specificity	Positive predictive value	Negative predictive value	Accuracy	Balanced accuracy
Original pre-operative FFTree without adjustment	0.47	0.51	0.09	0.90	0.51	0.49
Original pre-operative FFTree with threshold adjustment	0.25	0.83	0.13	0.92	0.78	0.54
Original peri-operative FFTree without adjustment	0.10	0.95	0.17	0.91	0.87	0.53
Original peri-operative FFTree with threshold adjustment	0.65	0.54	0.13	0.94	0.55	0.59

### Comparison of algorithm performance

#### Pre-operative algorithms

**Fig. 1:**
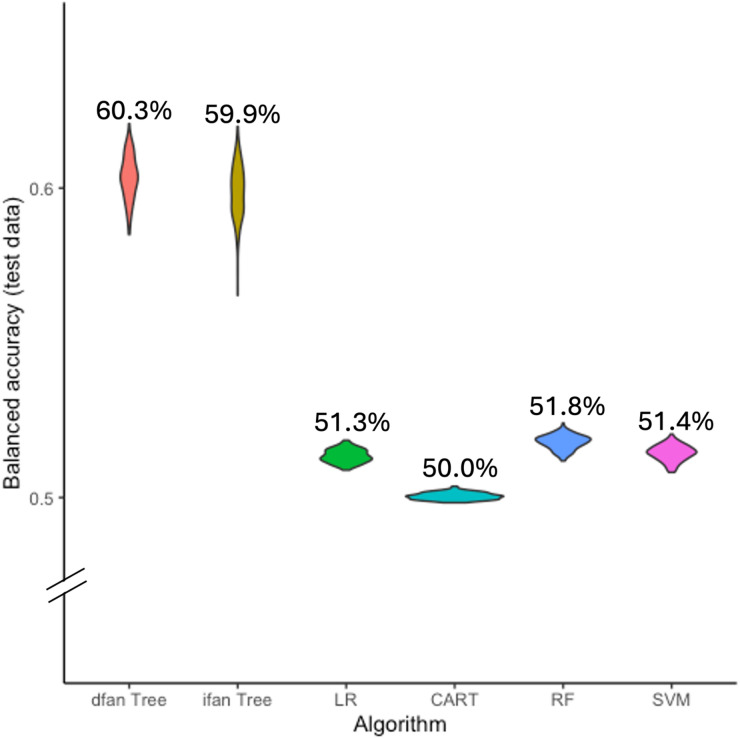
Bootstrap of balanced accuracy of all pre-operative algorithms on untrained test data. | *Note* Comparing fast-and-frugal trees established with the dfan (dfan Tree) and ifan (ifan Tree) algorithms with logistic regression (LR), classification and regression tree (CART), random forest (RF), and support vector machine (SVM) algorithms.

Performance of all models is summarized in Fig. 1; Table 2. The dfan and ifan FFTree algorithms showed the highest balanced accuracies (60.3% and 59.9%), followed by random forest (51.8%), SVM (51.4%), logistic regression (51.3%), and CART (50.0%). Overall differences between models were approximately 10% points.

**Table 2:** Test performance of pre-operative algorithms.

Algorithm	Sensitivity [95% CI]	Specificity [95% CI]	PPV [95% CI]	NPV [95% CI]	Accuracy [95% CI]	BACC [95% CI]
dfan	0.55	0.65	0.14	0.93	0.64	0.60
	[0.49; 0.65]	[0.54; 0.69]	[0.12; 0.15]	[0.93; 0.94]	[0.55; 0.68]	[0.58; 0.61]
ifan	0.57	0.61	0.13	0.93	0.61	0.59
	[0.47; 0.69]	[0.49; 0.70]	[0.12; 0.15]	[0.93; 0.94]	[0.50; 0.68]	[0.58; 0.61]
LR	0.03	0.997	0.50	0.91	0.91	0.51
	[0.02; 0.04]	[0.996; 0.998]	[0.39; 0.61]	[0.90; 0.91]	[0.90;0 0.91]	[0.51; 0.52]
CART	0.00	0.996	0.11	0.91	0.90	0.50
	[0.00; 0.01]	[0.995; 0.997]	[0.03; 0.25]	[0.90; 0.91]	[0.90; 0.91]	[0.50; 0.50]
RF	0.04	0.998	0.65	0.91	0.91	0.52
	[0.03; 0.05]	[0.997; 0.999]	[0.55; 0.76]	[0.91; 0.91]	[0.90; 0.91]	[0.51; 0.52]
SVM	0.03	0.999	0.71	0.91	0.91	0.51
	[0.02; 0.04]	[0.998; 0.999]	[0.61; 0.8]	[0.91; 0.91]	[0.90; 0.91]	[0.51; 0.52]

Values in brackets show bootstrapped 95% confidence intervals. The performance is shown for the dfan (dfan), ifan (ifan), logistic regression (LR), classification and regression tree (CART), random forest (RF), and support vector machine (SVM) algorithms. PPV = positive predictive value, NPV = negative predictive value, BACC = balanced accuracy.

Higher balanced accuracy of FFTrees was primarily driven by greater sensitivity, whereas comparator models showed lower sensitivity and less favorable sensitivity–specificity trade-offs (Table 2). FFTree performance also showed greater variability across bootstrap iterations, reflected in wider confidence intervals. Training performance of the more complex models, particularly random forest, exceeded test performance, indicating potential overfitting (Supplemental Table S1).

#### Peri-operative algorithms

**Fig. 2:**
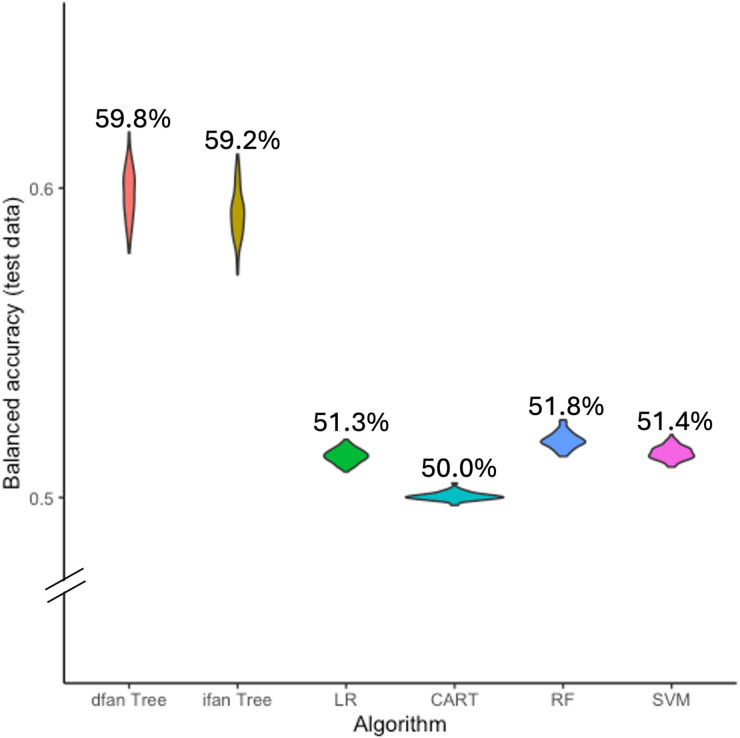
Bootstrap of balanced accuracy of all peri-operative algorithms on untrained test data. | *Note* Comparing fast-and-frugal trees established with the dfan (dfan Tree) and ifan (ifan Tree) algorithms with logistic regression (LR), classification and regression tree (CART), random forest (RF), and support vector machine (SVM) algorithms.

Performance results are shown in Fig. 2; Table 3. Incorporating peri-operative variables resulted in only marginal performance changes. The ifan FFTree achieved a balanced accuracy of 59.8%, similar to the pre-operative setting (59.2%). As in the pre-operative analysis, FFTrees achieved the highest balanced accuracy (~ 60%), followed by random forest (51.8%), SVM (51.4%), logistic regression (51.3%), and CART (50.0%).

**Table 3:** Test performance of perioperative algorithms.

Algorithm	Sensitivity [95% CI]	Specificity [95% CI]	PPV [95% CI]	NPV [95% CI]	Accuracy [95% CI]	BACC [95% CI]
dfan	0.62	0.59	0.13	0.94	0.59	0.60
	[0.49; 0.69]	[0.54; 0.68]	[0.13; 0.15]	[0.93; 0.94]	[0.55; 0.66]	[0.59; 0.62]
ifan	0.54	0.66	0.14	0.93	0.65	0.60
	[0.36; 0.69]	[0.49; 0.82]	[0.12; 0.17]	[0.92; 0.94]	[0.51; 0.77]	[0.58; 0.61]
LR	0.03	0.997	0.51	0.91	0.91	0.51
	[0.02; 0.04]	[0.996; 0.998]	[0.40; 0.65]	[0.90; 0.91]	[0.90; 0.91]	[0.51; 0.52]
CART	0.00	0.996	0.11	0.91	0.90	0.50
	[0; 0.01]	[0.995; 0.997]	[0.04; 0.2]	[0.90; 0.91]	[0.90; 0.91]	[0.5; 0.5]
RF	0.04	0.998	0.69	0.91	0.91	0.52
	[0.03; 0.05]	[0.997; 0.999]	[0.58; 0.8]	[0.90; 0.91]	[0.90; 0.91]	[0.51; 0.52]
SVM	0.03	0.999	0.71	0.91	0.91	0.51
	[0.02; 0.04]	[0.998; 0.999]	[0.58; 0.84]	[0.90; 0.91]	[0.90; 0.91]	[0.51; 0.52]

Values in brackets show bootstrapped 95% confidence intervals. The performance is shown for the dfan (dfan), ifan (ifan), logistic regression (LR), classification and regression tree (CART), random forest (RF), and support vector machine (SVM) algorithms. PPV = positive predictive value, NPV = negative predictive value, BACC = balanced accuracy.

Differences between models remained approximately 10% points. Variability in FFTree performance increased in the peri-operative setting, again reflected in wider confidence intervals. More complex models continued to show higher training than test performance, suggesting overfitting (Supplemental Table S1).

### Updated FFTrees based on the routine clinical dataset

**Fig. 3:**
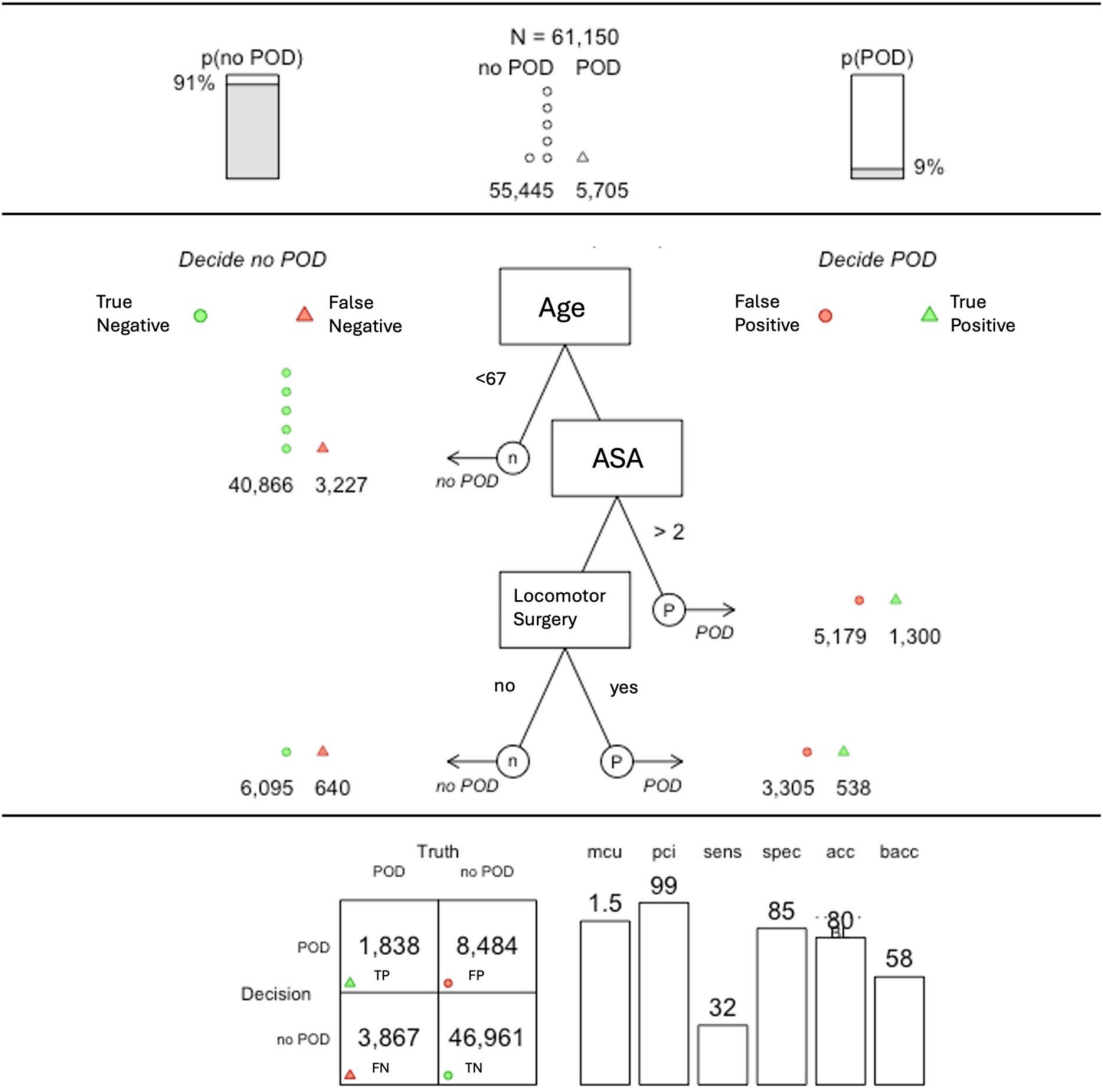
Updated fast-and-frugal decision tree for the pre-operative setting based on the routine clinical dataset. *Note* POD = postoperative delirium, ASA = American Society of Anesthesiologists Physical Status Classification, TP = true positive, FP = false positive, FN = false negative, TN = true negative, mcu = mean cues used, pci = percent information used, sens = sensitivity, spec = specificity, acc = accuracy, BL = baseline accuracy, bacc = balanced accuracy.

**Fig. 4:**
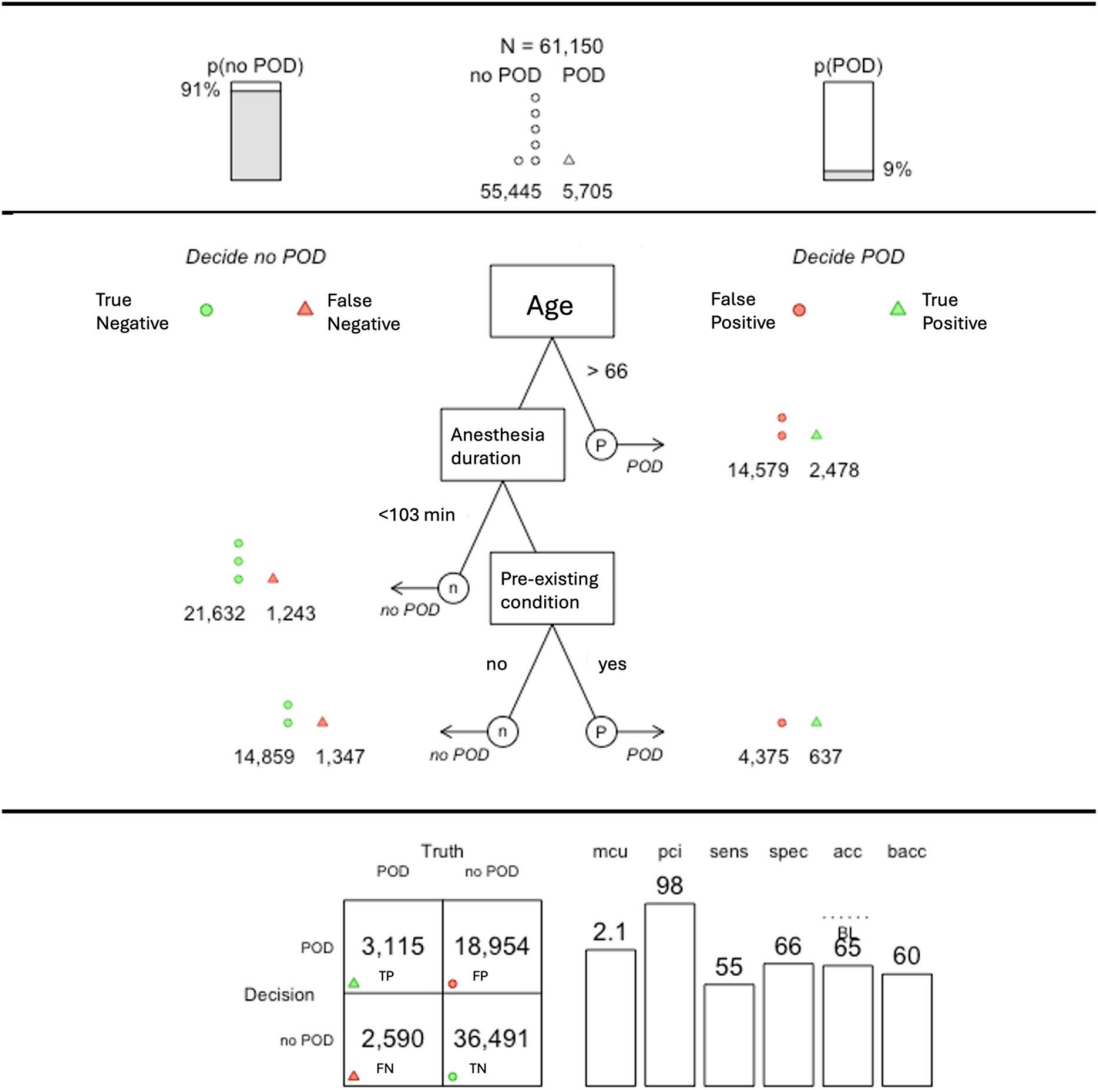
Updated FFTree for the peri-operative setting based on the routine clinical dataset. *Note* POD = postoperative delirium, TP = true positive, FP = false positive, FN = false negative, TN = true negative, mcu = mean cues used, pci = percent information used, sens = sensitivity, spec = specificity, acc = accuracy, BL = baseline accuracy, bacc = balanced accuracy.

Based on these results, two FFTrees were developed for routine clinical application: one for the pre-operative and one for the peri-operative setting (Figs. 3 and 4). Models were selected from multiple candidate trees (see Supplement) based on parsimony and practical applicability. Specifically, when several tree variants showed similar predictive performance, preference was given to models requiring fewer cues, as these allow for more efficient and easily implementable decision-making in routine clinical settings.

#### Pre-operative setting

The final pre-operative FFTree included three cues—age, ASA status, and locomotor surgery—and achieved a sensitivity of 32%, specificity of 85%, and balanced accuracy of 58% (Fig. 3). On average, only 1.5 cues were required per decision, corresponding to 99% information reduction. Alternative tree variants with different sensitivity–specificity trade-offs are provided in Supplemental Table S2.

#### Peri-operative setting

The peri-operative FFTree included age, anesthesia duration, and pre-existing conditions, achieving a sensitivity of 55%, specificity of 66%, and balanced accuracy of 60% (Fig. 4). On average, 2.1 cues were required per decision (98% information reduction). Alternative variants are shown in Supplemental Table S3.

## Discussion

The aim of this study was to evaluate whether Fast-and-Frugal Trees (FFTrees), previously developed using structured research data, remain applicable when transferred to routine clinical data. Using a large electronic health record dataset of more than 61,000 surgical patients, we assessed their transferability, compared them with commonly used machine-learning approaches, and developed updated FFTrees tailored to routine clinical practice.

FFTrees retained moderate predictive performance when applied to routine clinical data, despite substantial differences in data structure and quality compared to research datasets. As expected, performance decreased when applying previously published trees without modification but improved after recalibration of cue thresholds. This highlights both the challenges and feasibility of translating predictive models from controlled research settings to real-world clinical environments.

The reduction in performance likely reflects structural characteristics of routine clinical data, including incomplete documentation and the need to reconstruct key predictors from proxy variables. Such limitations are inherent to electronic health records and emphasize the importance of evaluating predictive tools under realistic clinical conditions. Improving the quality of routine clinical data may further enhance the performance and applicability of predictive models. Potential strategies include more standardized documentation of key risk factors (e.g., frailty, cognitive status), structured integration of routinely assessed clinical scores into electronic health records, and improved completeness of perioperative data entry. In addition, automated data capture and harmonized coding practices may reduce variability and missingness, thereby supporting more reliable risk prediction in routine clinical settings.

Across all models, predictive performance remained moderate, and differences between algorithms were small. This suggests that, in routine datasets characterized by heterogeneous and partially missing information, increasing model complexity does not necessarily result in meaningful gains in predictive accuracy. Rather than indicating general superiority of FFTrees, these findings highlight that model performance is context-dependent, with simpler models performing comparably when data quality is limited.

The predictors consistently selected by the updated FFTrees—particularly age, ASA status, anesthesia duration, and pre-existing medical conditions—are well-established risk factors for postoperative delirium. Their prominence can be interpreted mechanistically: advanced age reflects increased vulnerability of the central nervous system, ASA status captures overall physiological burden and comorbidity, and longer anesthesia duration may indicate prolonged exposure to perioperative stressors and neurocognitive strain. Together, these findings suggest that a small set of robust clinical indicators captures much of the predictive signal available in routine data.

Potential sources of bias should also be considered. The relatively low prevalence of postoperative delirium (9%) introduces class imbalance, which may affect sensitivity–specificity trade-offs. In addition, more complex models, particularly random forests, showed indications of overfitting, as reflected by discrepancies between training and test performance. These factors may partly explain why more complex algorithms did not outperform simpler approaches in this setting.

In this context, the primary contribution of FFTrees lies not in maximizing predictive accuracy but in providing a simple, transparent, and easily memorizable decision structure that supports clinicians in structuring risk assessment in routine clinical practice, where risk assessment often remains unsystematic and resources for intensive monitoring are limited. By reducing decision-making to a small number of sequential cues, FFTrees allow clinicians to reach a risk assessment after evaluating only limited information and without computational support. From this perspective, FFTrees should be understood less as high-precision predictive instruments and more as cognitive decision aids that facilitate consistent and structured risk factor assessment. Despite these risk factors being well established, they are often not applied or not arranged optimally in practice. FF trees can support medical decision-making by making an optimal cue structure explicit, depending on whether the goal is balanced accuracy or a more sensitive or specific outcome (also see Supplement). Their transparency may additionally support communication with patients and families and enable shared decision-making. FFTrees are not intended to replace comprehensive clinical assessment or more complex predictive models where these are feasible, but rather to support risk structuring in resource-constrained routine settings.

### Limitations

Several limitations should be considered when interpreting the findings of this study. First, the dataset was derived from routine clinical documentation rather than structured research data. As a result, some predictors were unavailable or had to be reconstructed from proxy variables (e.g., Charlson Comorbidity Index), which may have reduced model fidelity. Missing data were handled using mean and mode imputation, which may underestimate uncertainty and introduce bias. Second, cardiovascular and craniotomy surgeries were excluded. These patient groups are typically considered uniformly high-risk for postoperative delirium and are routinely managed with intensive monitoring and prevention protocols. As such, risk prediction is less likely to influence clinical decision-making in these settings. Our analysis therefore focused on patient populations in which delirium risk is less clearly defined and where risk stratification may meaningfully support the allocation of limited monitoring resources. However, this limits generalizability to high-risk surgical populations. Third, restricting the analysis to the last surgical intervention per patient may introduce selection bias, as patients undergoing multiple procedures could differ systematically from those with a single intervention. This approach may therefore affect the representativeness of the sample and should be considered when interpreting the findings. Fourth, the study was conducted within a single hospital system, which may limit transferability to other clinical settings with different patient populations and documentation practices. Fifth, predictive performance across all models remained moderate. Importantly, the aim of this study was not solely to maximize predictive accuracy, but to evaluate whether a simple, easily memorizable decision structure can support clinicians in structuring risk assessment in routine practice. In this context, even moderate performance may be clinically meaningful if it improves the consistency and feasibility of delirium risk evaluation. Finally, the study does not assess the impact of FFTrees on clinical workflows or patient outcomes, which should be addressed in future research.

## Conclusion

This study demonstrates that FFTrees can be a practical and easily implemented diagnostic tool for adhering to POD risk assessment guidelines. The simplicity of FFTrees offers several advantages. They are inherently robust to challenges such as overfitting and missing data, enabling them to achieve diagnostic performance comparable to more complex models. Their transparent, rule-based structure also makes them intuitive and accessible, facilitating effective communication with patients and novice practitioners alike. Furthermore, FFTrees are highly practical for everyday clinical use, as their straightforward design fosters rapid decision-making in dynamic treatment environments without requiring extensive computational resources or specialized training.

## Supplementary Information

Below is the link to the electronic supplementary material.

Supplementary Material 1

## Data Availability

Due to German data protection and patient privacy laws, we cannot provide the original data. We added comprehensive summary statistics to describe our raw data. For requesting the underlying raw data, please contact the ethics committee at Charité via Ethikkommission@charite.de. Odette Wegwarth and Helge Giese had full access to all the data in the study and take responsibility for the integrity of the data and the accuracy of the data analysis.
