# Supplementary material for: Translating predictive models into clinical practice: fast-and-frugal trees for postoperative delirium using routine data
Source: Sci Rep. 2026 Apr 18;16:12731. doi: 10.1038/s41598-026-47452-3 (PMC13091790; doi:10.1038/s41598-026-47452-3)
Supplement: Supplementary file 1 — Supplementary Material 1 [file 41598_2026_47452_MOESM1_ESM.docx]

Supplement: **Translating Predictive Models into Clinical Practice: Fast-and-Frugal Trees for Postoperative Delirium Using Routine Data**

Odette Wegwarth, Felix Balzer, Sebastian D. Boie, Niklas Giesa, Anika Müller, Jan K. Woike, Claudia Spies, & Helge Giese

Supplemental Table 1. Balanced accuracy performance for training data.

| pre-operative balanced accuracy | | | |
| --- | --- | --- | --- |
| Algorithm | M | LL | UL |
| dfan | 60.0% | 59.5% | 60.5% |
| ifan | 59.4% | 59.0% | 60.1% |
| LR | 52.1% | 51.9% | 52.3% |
| CART | 51.6% | 51.5% | 51.7% |
| RF | 60.8% | 60.4% | 61.3% |
| SVM | 51.6% | 51.5% | 51.7% |
| peri-operative balanced accuracy | | | |
| Algorithm | M | LL | UL |
| dfan | 60.8% | 60.3% | 61.2% |
| ifan | 60.0% | 59.5% | 60.5% |
| LR | 52.1% | 51.9% | 52.3% |
| CART | 51.6% | 51.5% | 51.7% |
| RF | 64.5% | 64.0% | 65.2% |
| SVM | 51.6% | 51.5% | 51.8% |

Notes. Comparing fast and frugal trees established with the dfan and ifan algorithms with logistic regression (LR), classification and regression tree (CART), random forest (RF), and support vector machine (SVM) algorithms. LL and UL denote the 95% bootstrapped confidence intervals.

Supplemental Table 2. Pre-operative tree variants.

| tree | sens | spec | ppv | npv | acc | nodes | cues | directions | thresholds | exits |
| --- | --- | --- | --- | --- | --- | --- | --- | --- | --- | --- |
| 1 | 0.52 | 0.68 | 0.14 | 0.93 | 0.67 | 4 | age; no preex. cond.; history NUDESC; ASA | >;>;>;> | 66;0;0;2 | 1;0;1;0.5 |
| 2 | 0.45 | 0.74 | 0.15 | 0.93 | 0.71 | 3 | age; no preex. cond.; history NUDESC | >;>;> | 66;0;0 | 1;0;0.5 |
| 3 | 0.37 | 0.81 | 0.17 | 0.93 | 0.77 | 4 | age; ASA ; l.motor surgery; age | >;>;=;> | 66;2;1;77 | 0;1;1;0.5 |
| 4 | 0.32 | 0.85 | 0.18 | 0.92 | 0.8 | 3 | age; ASA ; l.motor surgery | >;>;= | 66;2;1 | 0;1;0.5 |
| 5 | 0.89 | 0.25 | 0.11 | 0.96 | 0.31 | 5 | age; no preex. cond.; birth; ASA ; hearing s. surgery | >;>;=;>;= | 66;0;0;1;0 | 1;1;0;0;0.5 |
| 6 | 0.93 | 0.17 | 0.1 | 0.96 | 0.24 | 5 | age;no preex. cond.; birth; ASA ; l.motor surgery | >;>;=;>;= | 66;0;0;1;1 | 1;1;0;1;0.5 |
| 7 | 0.1 | 0.97 | 0.27 | 0.91 | 0.89 | 3 | age; ASA ; age | >;>;> | 66;2;79 | 0;0;0.5 |
| 8 | 0.99 | 0.06 | 0.1 | 0.99 | 0.15 | 3 | age; no preex. cond.; birth | >;>;= | 66;0;0 | 1;1;0.5 |

The FFTree variants presented here in the Supplement allow clinicians to select a decision rule based on the desired balance between sensitivity (identifying patients at risk of postoperative delirium) and specificity (avoiding unnecessary monitoring of low-risk patients).

In clinical practice, the choice of an FFTree should be guided by the local clinical context and resource availability. If the priority is to minimize missed cases of delirium (e.g., in high-risk environments), trees with higher sensitivity may be preferred, accepting a higher number of false positives and increased monitoring. Conversely, in settings with limited resources, trees with higher specificity may be selected to reduce unnecessary monitoring while accepting a higher risk of missed cases. Thus, the FFTree framework does not provide a single “optimal” model but rather a set of transparent decision rules that can be tailored to different clinical priorities. The selected tree can be implemented directly as a simple sequential decision rule (e.g., “If age > X → high risk; else if ASA > Y → high risk; otherwise low risk”), enabling rapid and consistent risk assessment without computational support.

Supplemental Table 3. Peri-operative tree variant

| tree | nodes | sens | spec | ppv | npv | acc | cues | directions | thresholds | exits |
| --- | --- | --- | --- | --- | --- | --- | --- | --- | --- | --- |
| 1 | 4 | 0.66 | 0.56 | 0.13 | 0.94 | 0.57 | age; anesthesia duration; no preex. cond.; anesthesia duration | >;>;>;> | 66;102.95;0;168 | 1;0;1;0.5 |
| 2 | 3 | 0.55 | 0.66 | 0.14 | 0.93 | 0.65 | age; anesthesia duration; no preex. cond. | >;>;> | 66;103;0 | 1;0;0.5 |
| 3 | 5 | 0.75 | 0.44 | 0.12 | 0.95 | 0.47 | age; anesthesia duration; no preex. cond.; anesthesia duration; ASA | >;>;>;>;> | 66;102.95;0;168;1 | 1;0;1;1;0.5 |
| 4 | 3 | 0.36 | 0.83 | 0.18 | 0.93 | 0.78 | age; ASA; anesthesia duration | >;>;> | 66;2;118 | 0;1;0.5 |
| 5 | 5 | 0.82 | 0.35 | 0.12 | 0.95 | 0.4 | age; anesthesia duration; birth; anesthesia duration; no preex. cond. | >;>;=;>;> | 66;103;0;60;0 | 1;1;0;0;0.5 |
| 6 | 5 | 0.95 | 0.16 | 0.10 | 0.97 | 0.23 | age; anesthesia duration; birth; anesthesia duration; respiratory surgery | >;>;=;>;= | 66;102.95;0;60;1 | 1;1;0;1;0.5 |
| 7 | 3 | 0.15 | 0.95 | 0.25 | 0.92 | 0.88 | age; ASA ; anesthesia duration | >;>;> | 66;2;112 | 0;0;0.5 |
| 8 | 3 | 0.99 | 0.06 | 0.10 | 0.99 | 0.15 | age; anesthesia duration; birth | >;>;= | 66;103;0 | 1;1;0.5 |

The FFTree variants presented here in the Supplement allow clinicians to select a decision rule based on the desired balance between sensitivity (identifying patients at risk of postoperative delirium) and specificity (avoiding unnecessary monitoring of low-risk patients).

In clinical practice, the choice of an FFTree should be guided by the local clinical context and resource availability. If the priority is to minimize missed cases of delirium (e.g., in high-risk environments), trees with higher sensitivity may be preferred, accepting a higher number of false positives and increased monitoring. Conversely, in settings with limited resources, trees with higher specificity may be selected to reduce unnecessary monitoring while accepting a higher risk of missed cases. Thus, the FFTree framework does not provide a single “optimal” model but rather a set of transparent decision rules that can be tailored to different clinical priorities. The selected tree can be implemented directly as a simple sequential decision rule (e.g., “If age > X → high risk; else if ASA > Y → high risk; otherwise low risk”), enabling rapid and consistent risk assessment without computational support.
